# Increased migraine risk in osteoporosis patients: a nationwide population-based study

**DOI:** 10.1186/s40064-016-3090-8

**Published:** 2016-08-22

**Authors:** Chieh-Hsin Wu, Zi-Hao Zhang, Ming-Kung Wu, Chiu-Huan Wang, Ying-Yi Lu, Chih-Lung Lin

**Affiliations:** 1 Department of Dermatology, Kaohsiung Veterans General Hospital, No. 386 Dazhong 1st Rd, Kaohsiung, 81362 Taiwan; 2 Cosmetic Applications and Management Department, Yuh-Ing Junior College of Health Care and Management, Kaohsiung, Taiwan; 3 Graduate Institute of Medicine, College of Medicine, Kaohsiung Medical University, Kaohsiung, Taiwan; 4 Department of Neurosurgery, Kaohsiung Medical University Hospital, Kaohsiung Medical University, No. 100 Tzyou 1st Road, Kaohsiung, 80708 Taiwan; 5 Department of Neurosurgery, The No. 7 People’s Hospital of Hebei Province, Dingzhou, 073000 Hebei People’s Republic of China; 6 Department of Psychiatry, Kaohsiung Chang Gung Memorial Hospital and Chang Gung University College of Medicine, Kaohsiung, 807 Taiwan; 7 Department of Nursing, Kaohsiung Medical University Hospital, Kaohsiung Medical University, No. 100 Tzyou 1st Road, Kaohsiung, 80708 Taiwan; 8 Department of Neurosurgery, Faculty of Medicine, College of Medicine, Kaohsiung Medical University, Kaohsiung, Taiwan

**Keywords:** Osteoporosis, Migraine, Nationwide population-based study

## Abstract

**Background:**

Osteoporosis and migraine are both important public health problems and may have overlapping pathophysiological mechanisms. The aim of this study was to use a Taiwanese population-based dataset to assess migraine risk in osteoporosis patients.

**Methods:**

The Taiwan National Health Insurance Research Database was used to analyse data for 40,672 patients aged ≥20 years who had been diagnosed with osteoporosis during 1996–2010. An additional 40,672 age-matched patients without osteoporosis were randomly selected as the non-osteoporosis group. The relationship between osteoporosis and migraine risk was estimated using Cox proportional hazard regression models.

**Results:**

During the follow-up period, 1110 patients with osteoporosis and 750 patients without osteoporosis developed migraine. After controlling for covariates, the overall incidence of migraine was 1.37-fold higher in the osteoporosis group than in the non-osteoporosis group (3.72 vs. 1.24 per 1000 person-years, respectively). Migraine risk factors included high Charlson Comorbidity Index score, female gender, hypertension, depression, asthma, allergic rhinitis, obesity, and tobacco use disorder.

**Conclusions:**

Our results indicate that patients with a history of osteoporosis had a higher risk of migraine.

## Background

Both osteoporosis and migraine are common conditions that can affect quality of life and can impose large social and economic burdens (Kuo et al. 2015; Manandhar et al. 2015a, b; Mbewe et al. 2015; Rao et al. 2015; Steiner et al. 2015; Lampl et al. 2016). The National Institutes of Health Consensus Development Conference Statement defines osteoporosis as a skeletal disorder characterized by diminished bone strength resulting in increased fracture risk. Bone strength is measured in terms of both bone mineral density (BMD) and bone quality (Nih Consensus Development Panel on Osteoporosis Prevention and Therapy 2001). In elderly populations, osteoporosis affects approximately 30 % of women and 12 % of men (Rachner et al. 2011). Migraine is a neurological disorder that manifests as a debilitating headache associated with altered sensory perception (Goadsby et al. 2002; Charles 2013; Baykan et al. 2015; Manandhar et al. 2015a, b). The International Headache Society defines migraine as a headache that lasts for 4–72 h and has at least two of the following characteristics: pulsating quality, unilateral localization, moderate-to-severe pain intensity, and aggravation by movement (Headache Classification Subcommittee of the International Headache 2004). Previous studies have identified interacting relationships among migraine, various sleep disorders, depression, psoriasis, restless legs syndrome and cardiovascular disease (Kelman and Rains 2005; Pompili et al. 2009; Schurks et al. 2009; Cho et al. 2015; Egeberg et al. 2015; Kim et al. 2016; Risal et al. 2016). Migraine is associated with episodes of local sterile meningealinflammation, hypersensitized pain pathways, and increased inflammatory cytokines that contribute to the pathogenesis of osteoporosis, such as interleukins (ILs) or tumor necrosis factor-α (Braun and Schett 2012; Egeberg et al. 2015). Like osteoporosis and other inflammatory conditions, migraine is also apparently associated with systemic endothelial dysfunction (Vanmolkot et al. 2007; Sacco et al. 2013; Steyers and Miller 2014). Although no recent studies have suggested a link between osteoporosis and migraine, both conditions are independent risk factors for cardiovascular disease (Sumino et al. 2008; Bigal et al. 2010; Hyder et al. 2010), and both are comorbid with pain-related and psychiatric conditions (Kalaydjian and Merikangas 2008; Radaei et al. 2014). We therefore investigated the impact of osteoporosis on migraine risk in a nationwide cohort in Taiwan.

## Methods

### Database

This population-based cohort study used data obtained from the Taiwan National Health Insurance Research Database (NHIRD) maintained by the national health care system of Taiwan. The NHIRD is an encrypted secondary database containing medical data for approximately 99 % of the 23.74 million residents of Taiwan. The Taiwan national health insurance program allows researchers to access this database of administrative data for patients. The NHIRD files are composed of comprehensive use and enrollment information of the patients. Besides, under regulations of the Personal Electronic Data Protection Law of Taiwan, all citizens and hospital identities in the NHIRD database were decoded. This retrospective cohort study analysed 1996–2010 data contained in a subset of the NHIRD, the Longitudinal Health Insurance Database 2010, which comprises data for 1 million beneficiaries randomly sampled from the primary NHIRD. In this study, diseases were identified and classified according to the diagnostic codes of the International Classification of Diseases, Ninth Revision, Clinical Modification (ICD-9-CM).

### Ethical approval

The study was performed in accordance with the Declaration of Helsinki guidelines and was also evaluated and approved by the Institutional Review Board of Kaohsiung Medical University Hospital (KMUHIRB-EXEMPT (II)-20160016).

### Study population

The study cohort included 40,672 patients aged 20 years or older who had been diagnosed with osteoporosis (ICD-9-CM code 733.0) during 1996–2010. To maximize accuracy, the analysis was limited to patients who had a record of ≥2 osteoporosis diagnoses during ambulatory visits or ≥1 diagnoses during inpatient care, an ICD-9 code assigned by an orthopaedist, and at least one BMD examination. The index date was defined as the date of the first clinical visit for osteoporosis. A migraine (ICD-9-CM code 346) was defined as a history of two or more migraine diagnoses in ambulatory visits or one or more migraine diagnoses in inpatient care as well as a record of an ICD-9 code for migraine assigned by a neurologist. The date of diagnosis was defined as the date of the first diagnosis of osteoporosis. Matching control subjects were assigned an identical “pseudo date of diagnosis”, which was the date of diagnosis in matched cases. The exclusion criteria were diagnosis of migraine before or on the index date, incomplete information, and age younger than 20 years.

**Fig. 1:**
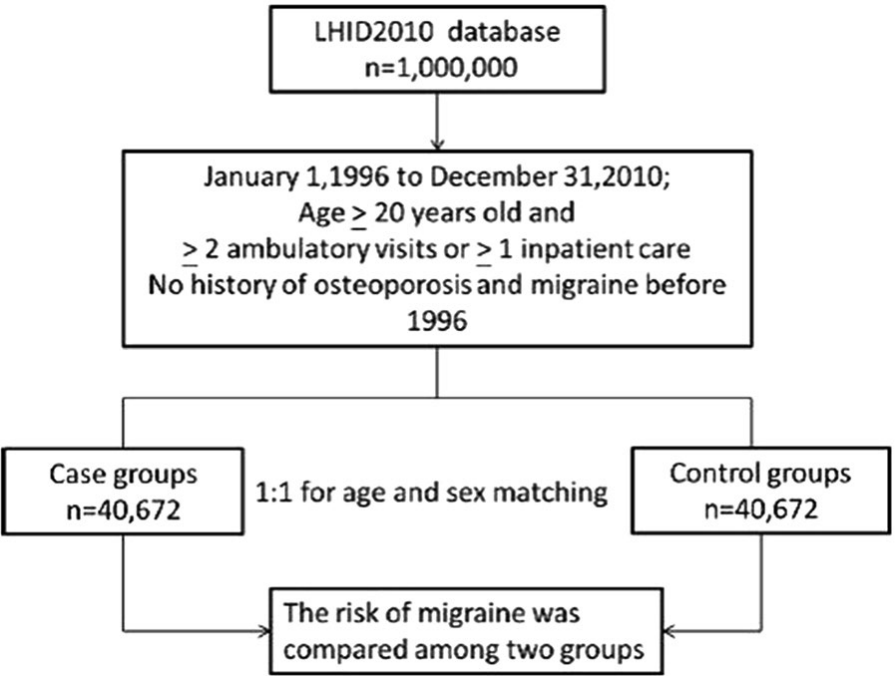
Flow diagram of the present study from the National Health Insurance Research Database in Taiwan. *LHID* longitudinal Health Insurance Database

The ratio of osteoporosis to non-osteoporosis patients was kept at 1:1 to enhance the power of statistical tests and to obtain a sufficient number of migraine cases for stratified analyses. The patients in the non-osteoporosis cohort were selected by a simple random sampling method in which one insured patient without osteoporosis was randomly selected and frequency matched with each person in the osteoporosis cohort in the same period by age, gender, and year of osteoporosis diagnosis. A post hoc sample size was calculated to determine statistical power. Logistic regression analysis was used to obtain a 4-digit match of the propensity score for each patient with the covariates, including age and gender. As a result, 40,672 subjects were enrolled in the non-osteoporosis cohort. Figure 1 shows a flowchart of the study procedure.

### Outcome and comorbidities

The patients in both the osteoporosis and non-osteoporosis cohorts were followed up until diagnosis with migraine, withdrawal from insurance, or the end of 2010, whichever occurred first. Baseline comorbidities identified by ICD-9-CM codes in the claims records before the index date included hypertension (ICD-9-CM codes 401-405), diabetes mellitus (ICD-9-CM code 250), hyperlipidemia (ICD-9-CM code 272), depression (ICD-9-CM codes 296.2, 296.3, 300.4 and 311), asthma (ICD-9-CM code 493), allergic rhinitis (ICD-9-CM code 477), psoriasis (ICD-9-CM code 696.1), obesity (ICD-9-CM code 278), tobacco use disorder (ICD-9-CM code 350.1), and alcohol attributed disease (ICD-9-CM codes 291.0-9, 303, 305.0, 357.5, 425.5, 535.3, 571.0-3, 980.0 and V11.3). The Charlson Comorbidity Index (CCI) score was used to assess the severity of comorbidities, i.e., myocardial infarction, congestive heart failure, peripheral vascular disease, cerebrovascular disease, dementia, chronic pulmonary disease, rheumatic disease, peptic ulcer disease, liver disease (mild, moderate, or severe), diabetes (with or without chronic complication), hemiplegia or paraplegia, renal disease, any malignancy (including lymphoma and leukemia but excluding skin malignancy), metastatic solid tumor, human immunodeficiency virus infection and acquired immune deficiency syndrome. The CCI scores were then categorized into four levels: 0, 1–2, 3–4 and ≥5.

### Statistical analyses

Chi square test was used to compare distributions of categorical demographics and clinical characteristics between the osteoporosis and non-osteoporosis cohorts. The Student t test and Wilcoxon rank-sum test were used as appropriate to compare mean age and follow-up time (y) between the two cohorts. The Kaplan–Meier method was used to estimate cumulative incidence, and the differences between the curves were tested by 2-tailed log-rank test. For osteoporosis patients, survival was calculated until hospitalization, an ambulatory visit for migraine, or the end of the study period (December 31, 2010), whichever occurred first. Incidence rates of migraine estimated in 1000 person-years were compared between the two cohorts. Univariable and multivariable Cox proportional hazard regression models were used to calculate hazard ratios (HRs) and 95 % confidence intervals (CIs) for migraine if the proportional hazards assumption was satisfied. The multivariable Cox models were adjusted for age, gender, CCI score, and relevant comorbidities. A 2-tailed *P* value of <0.05 was considered statistically significant. All data processing and statistical analyses were performed using Statistical Analysis Software, version 9.4 (SAS Institute, Cary, NC, USA).

## Results

### Baseline characteristics of patients with and without osteoporosis

The baseline demographic characteristics and comorbidities in the two cohorts are presented in Table 1. In the osteoporosis cohort, 82.48 % patients were female. Compared to the non-osteoporosis cohort, the osteoporosis cohort had significantly higher percentages of patients with hypertension (70.12 vs. 54.07, P < 0.001), diabetes mellitus (38.58 vs. 27.71, P < 0.001), hyperlipidemia (61.28 vs. 43.96, P < 0.001), depression (21.21 vs. 11.71, P < 0.001), asthma (28.77 vs. 19.20, P < 0.001), allergic rhinitis (44.62 vs. 32.38, P < 0.001), psoriasis (2.15 vs. 1.43, P < 0.001), obesity (2.64 vs. 2.11, P < 0.001), tobacco use disorder (1.91 vs. 1.04, P < 0.001) and alcohol-attributable disease (2.63 vs. 1.92, P < 0.001). The osteoporosis cohort also had higher CCI scores. During a median observation time of 3.5 years, 2.73 % (1110) of the osteoporosis patients had migraine (interquartile range [IQR] 1.5–6.2). The migraine incidence of osteoporosis cohort was significantly (P < 0.001) higher than that in the non-osteoporosis patients (751 with migraine out of 40,672 age- and gender-matched controls [1.85 %]) during a median observation time of 7.2 years [IQR 4.8–10.5]). Migraine development was significantly faster in the osteoporosis group (3.5 years) compared to the non-osteoporosis group (7.2 years) for the respective observation periods.

**Table 1:** Baseline characteristics of patients with and without osteoporosis

Variables	Osteoporosis		*P* value
	Yes (N = 40,672)	No (N = 40,672)	
Migraine patients, n (%)	1110 (2.73)	751 (1.85)	<0.001
Period of developing migraine, median (IQR), years	3.5 (1.5–6.2)	7.2 (4.8–10.5)	<0.001
Mean age at diagnosis of migraine, years	57.9 (10.4)	62.6 (10.3)	<0.001
Age group, n (%)			
20–49	7361 (18.10)	7361 (18.10)	
≥50	33,311 (81.90)	33,311 (81.90)	1.000
Gender, n (%)			
Men	7125 (17.52)	7125 (17.52)	
Women	33,547 (82.48)	33,547 (82.48)	1.000
Charlson Comorbidity Index, n (%)			
0	1912 (4.70)	7744 (19.04)	
1–2	9667 (23.77)	14,437 (35.50)	
3–4	11,911 (29.29)	9861 (24.25)	
≥5	17,182 (42.25)	8630 (21.22)	<0.001
Co-morbidity, n (%)			
Hypertension	28,521 (70.12)	21,991 (54.07)	<0.001
Diabetes mellitus	15,693 (38.58)	11,272 (27.71)	<0.001
Hyperlipidemia	24,923 (61.28)	17,879 (43.96)	<0.001
Depression	8625 (21.21)	4764 (11.71)	<0.001
Asthma	11,700 (28.77)	7807 (19.20)	<0.001
Allergic rhinitis	18,146 (44.62)	13,168 (32.38)	<0.001
Psoriasis	874 (2.15)	583 (1.43)	<0.001
Obesity	1073 (2.64)	857 (2.11)	<0.001
Tobacco use disorder	776 (1.91)	422 (1.04)	<0.001
Alcohol attributed disease	1069 (2.63)	780 (1.92)	<0.001

*IQR* interquartile range, *SD* standard deviation

### Migraine incidence and risk

The migraine incidence and HRs by gender, age and comorbidity are stratified in Table 2. During the follow-up period, migraine developed in 2.73 % (1110) of the osteoporosis patients and in 1.85 % (750) of the non-osteoporosis patients. The overall migraine risk was 1.37 times greater in the osteoporosis group compared to the non-osteoporosis group (3.72 vs. 1.24 per 1000 person-years, respectively) after adjusting for age, gender, CCI, and related comorbidities (hypertension, diabetes mellitus, hyperlipidemia, depression, asthma, allergic rhinitis, psoriasis, obesity, tobacco use disorder and alcohol-attributable disease).

**Table 2:** Incidence and hazard ratios of migraine by demographic characteristics and comorbidity among patients with or without osteoporosis

Variables	Patients with osteoporosis			Patients without osteoporosis			Compared to non- osteoporosis	
	Migraine	PYs	Rate	Migraine	PYs	Rate	CrudeHR^a^ (95 % CI)	Adjusted HR^a^ (95 % CI)
Overall	1110	324,126.20	3.42	751	604,550.99	1.24	2.48 (2.25–2.73)^c^	1.37 (1.23–1.51)^c^
Gender								
Men	104	45,604.93	2.28	78	106,313.43	0.73	2.80 (2.04–3.85)^c^	1.66 (1.19–2.31)^d^
Women	1006	278,521.27	3.61	573	498,237.56	1.35	2.39 (2.17–2.66)^c^	1.34 (1.19–1.49)^c^
Stratify by age								
20–49	252	64,474.34	3.91	130	109,506.02	1.19	2.97 (2.39–3.68)^c^	1.46 (1.17–1.82)^c^
≥50	858	259,651.83	3.30	621	495,044.97	1.25	2.37 (2.12–2.64)^c^	1.34 (1.20–1.50)^c^
Comorbidity^b^								
No	19	20,949.87	0.91	43	127,923.68	0.34	2.41 (1.40–4.14)^e^	1.78 (1.03–3.06)^f^
Yes	1091	303,176.33	3.59	708	476,627.31	1.49	2.18 (1.97–2.41)^c^	1.34 (1.22–1.49)^c^

*PYs* person-years, *Rate* incidence rate in per 1000 person-years, *95* *% CI* 95 % confidence interval, *HR* hazard ratio

^a^Model adjusted for age, gender, Charlson Comorbidity Index and relevant comorbidities (hypertension, diabetes mellitus, hyperlipidemia, depression, asthma, allergic rhinitis, psoriasis, obesity, tobacco use disorder and alcohol attributed disease)

^b^Patients with any examined comorbidities, including hypertension, diabetes mellitus, hyperlipidemia, depression, asthma, allergic rhinitis, psoriasis, obesity, tobacco use disorder and alcohol attributed disease, were classified as the comorbidity group

^c^P < 0.001

^d^P = 0.003

^e^P = 0.001

^f^P = 0.037

The gender-specific analyses showed that, in both cohorts, the incidence of osteoporosis was higher in women than in men (3.61 vs. 2.28 per 1000 person-years, respectively, in the osteoporosis cohort; 1.35 vs. 0.73 per 1000 person-years, respectively, in the non-osteoporosis cohort). Additionally, the osteoporosis group had a significantly higher migraine risk in both genders (adjusted HR 1.34, 95 % CI 1.19–1.49 for women; adjusted HR 1.66, 95 % CI 1.19–2.31 for men).

Although the incidence of migraine was consistently higher in all age groups in the osteoporosis cohort compared to the non-osteoporosis cohort, the migraine risk decreased with age. Age-specific risk comparisons showed that, compared to the non-osteoporosis cohort, the osteoporosis cohort had a significantly higher migraine risk in patients under 50 years old (adjusted HR 1.46, 95 % CI 1.17–1.82, P < 0.001) than in patients over 50 years old (HR 1.34, 95 % CI 1.20–1.50, P < 0.001). Regardless of comorbidities, migraine risk was higher in osteoporosis patients than in non-osteoporosis patients. However, the migraine risk contributed by osteoporosis decreased in the presence of comorbidity.

**Fig. 2:**
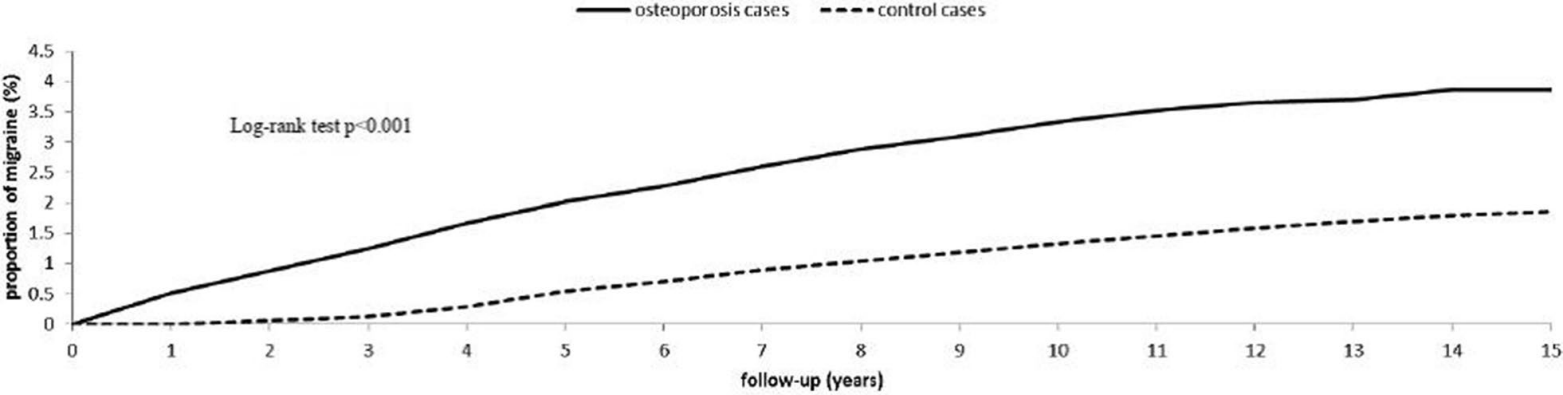
Cumulative incidence of migraine for adult patients with osteoporosis and the general population control cohort

The Kaplan–Meier curves for the cumulative incidence of migraine between the osteoporosis and non-osteoporosis groups at the 15-year follow up are compared in Fig. 2. The Kaplan–Meier curves showed a significantly higher cumulative incidence of migraine in the osteoporosis cohort compared to the non-osteoporosis cohort (log-rank test P < 0.001).

### Risks factors for migraine in osteoporosis patients

The Cox regression analysis revealed the following risk factors for migraine in the osteoporosis group: high CCI score, female gender, hypertension, depression, asthma, allergic rhinitis, obesity, and tobacco use disorder. Risk factors for migraine were female gender (adjusted HR 1.51, 95 % CI 1.23–1.85), hypertension (adjusted HR 1.19, 95 % CI 1.02–1.38), depression (adjusted HR 2.36, 95 % CI 2.09–2.66), asthma (adjusted HR 1.28, 95 % CI 1.13–1.45), allergic rhinitis (adjusted HR 1.46, 95 % CI 1.29–1.66), obesity (adjusted HR 1.79, 95 % CI 1.40–2.28), tobacco use disorder (adjusted HR 2.30, 95 % CI 1.78–2.97) and high CCI (adjusted HR 1.57, 95 % CI 1.44–1.71) (Table 3).

**Table 3:** Cox regression model: significant predictors of migraine after osteoporosis

Variables	Adjusted HR^a^	(95 % CI)	*P* value
Age	0.76	(0.72–0.81)	<0.001
Charlson Comorbidity Index	1.57	(1.44–1.71)	<0.001
Female gender	1.51	(1.23–1.85)	<0.001
Hypertension	1.19	(1.02–1.38)	0.027
Depression	2.36	(2.09–2.66)	<0.001
Asthma	1.28	(1.13–1.45)	0.001
Allergic rhinitis	1.46	(1.29–1.66)	<0.001
Obesity	1.79	(1.40–2.28)	<0.001
Tobacco use disorder	2.30	(1.78–2.97)	<0.001

The adjusted HR and 95 % CI were estimated by a stepwise the Cox proportional hazards regression method

*HR* hazard ratio, *95* *% CI* 95 % confidence interval

^a^Model adjusted for age, gender, Charlson Comorbidity Index and relevant comorbidities (hypertension, diabetes mellitus, hyperlipidemia, depression, asthma, allergic rhinitis, psoriasis, obesity, tobacco use disorder and alcohol attributed disease)

## Discussion

To our knowledge, this is the first nationwide population-based study of the relationship between osteoporosis and subsequent migraine in an Asian population. During the follow-up period, migraine developed in 2.73 % (1110) patients with osteoporosis and in 1.85 % (750) patients without osteoporosis. After controlling for potential confounding factors, migraine risk was 1.37-fold higher in the osteoporosis group than in the non-osteoporosis group. Patients with osteoporosis, particularly those with high CCI score, female gender, hypertension, depression, asthma, allergic rhinitis, obesity, and tobacco use disorder, had a high migraine risk.

The exact mechanisms underlying the relationship between migraine and osteoporosis are likely to be elusive. However, several lines of evidence in the literature suggest that osteoporosis and migraine have a shared pathophysiology. First, bone density is significantly associated with magnesium, an essential micronutrient with a wide range of metabolic, structural and regulatory functions (Jahnen-Dechent and Ketteler 2012). In humans, magnesium deficiency contributes to osteoporosis. Low serum magnesium is a co-contributing factor in osteopenia in adults with sickle cell anemia (Elshal et al. 2012). Moreover, an association between serum magnesium and bone density has been identified in pre- and post-menopausal women (Saito et al. 2004; Song et al. 2007). Magnesium deficiency also has a strong association with migraine attacks (Welch and Ramadan 1995). Gallai et al. showed that individuals suffering from migraine headaches had lower plasma and saliva magnesium levels between the attacks compared to controls without migraine headaches (Gallai et al. 1992). Both osteoporosis and migraine are associated with hypomagnesemia, which suggests an interplay between osteoporosis and migraine. Second, the relationship between migraine and osteoporosis might be explained at least partly by their common inflammatory mediators. Neurogenic inflammation resulting from activation of the trigeminal vascular system is the main cause of the pain produced by migraine. Stimulation of trigeminal ganglion nociceptors induces the release of proinflammatory substances, particularly calcitonin gene-related peptide (CGRP) (Silberstein 2004; Dalkara et al. 2006; D’Andrea and Leon 2010). Individuals with osteoporosis also had elevated CGRP levels (Lin et al. 2001). Experimental injections of CGRP into eviratated rats indicate that CGRP may also affect the release of osteoblastic cytokines and may indi-rectly regulate the suppressed bone resorption of osteoclasts (Valentijn et al. 1997). Inflammatory cytokines associated with osteoporosis such as tumor necrosis factor-αand IL-6 (Braun and Schett 2012; Wiseman et al. 2014) are elevated at the onset of migraine attacks (Perini et al. 2005). Finally, C-reactive protein, which increases during systemic inflammation, is elevated in both osteoporosis and migraine (Vanmolkot and de Hoon 2007; de Pablo et al. 2012). Thus, the inflammatory state caused by osteoporosis may increase the frequency or severity of migraine headaches by exacerbating the inflammatory response.

The strength of our study is the use of a large sample that is highly representative of the general population and provides sufficient statistical power to identify an association between osteoporosis and migraine risk. However, several limitations must be considered when interpreting these findings. One limitation is that the analysis only included observational data, i.e., ICD-9-CM codes, the accuracy of which depends on the clinical performance of individual physicians. That is, the accuracy of diagnostic codes in the database is a potential limitation. Notably, however, the Taiwan National Health Insurance program requires all insurance claims to be reviewed and audited by medical reimbursement specialists in the Bureau of National Health Insurance, which supports the validity and accuracy of the observed associations between osteoporosis and migraine. Furthermore, many studies have already used the NHIRD database because of its large size and long follow-up period (Chiu et al. 2015; Chu et al. 2015; Wu et al. 2015, 2016a, b, c, d). A second limitation is that the NHIRD does not contain detailed data that can be used to identify osteoporosis risk factors such as exercise capacity, body mass index, smoking, alcohol consumption, and dietary habits. A third limitation is that the Taiwan population analysed in this study was mostly of Chinese descent. Therefore, caution is needed when extrapolating the results to other ethnic groups; further studies are needed to determine whether these findings can be generalized to other ethnicities. Finally, this retrospective cohort study may have been biased by unrecognized or unadjusted confounding variables, despite the use of statistical methods for reducing their confounding effects.

## Conclusions

In summary, this nationwide population-based cohort study revealed that adult patients with osteoporosis had a significantly higher risk of developing subsequent migraine compared to controls without osteoporosis. Clinicians should be aware that osteoporosis is a potential risk factor for migraine. Further studies are recommended to confirm this association and to explore its mechanisms.

## Abbreviations

BMD: bone mineral density
CGRP: calcitonin gene-related peptide
CCI: Charlson Comorbidity Index
CI: confidence interval
HR: hazard ratio
ICD-9-CM: International Classification of Diseases, Ninth Revision, Clinical Modification
IL: interleukin
NHIRD: National Health Insurance Research Database
